# Increased miR-20b Level in High Grade Cervical Intraepithelial Neoplasia

**DOI:** 10.1007/s12253-020-00852-w

**Published:** 2020-07-08

**Authors:** Tímea Szekerczés, Ádám Galamb, Norbert Varga, Márta Benczik, Adrienn Kocsis, Krisztina Schlachter, András Kiss, Nándor Ács, Zsuzsa Schaff, Csaba Jeney, Gábor Lendvai, Gábor Sobel

**Affiliations:** 1 2nd Department of Pathology, Semmelweis University, Budapest, Hungary; 2 Department of Obstetrics and Gynecology, Semmelweis University, Üllői 78/A, Budapest, 1082 Hungary; 3 Nuffield Department of Clinical Neurosciences, Sleep & Circadian Neuroscience Institute, Oxford University, Oxford, UK; 4 SYNLAB Hungary Kft, Budapest, Hungary; 5 NEUMANN Diagnostics Ltd, Budapest, Hungary; 6 Present Address: Department of Pathology, National Institute of Oncology, Budapest, Hungary; 7 Department of Microsystems Engineering, Albert-Ludwigs University, Freiburg, Germany

**Keywords:** Cervical cancer, Cervical intraepithelial neoplasia (CIN), microRNA, Human papilloma virus

## Abstract

Cervical cancer is a common malignant tumor worldwide ranking fourth in incidence and mortality among females, which was reduced significantly by cytology screening and human papilloma virus (HPV) DNA testing. The specificity of cytology is high; however, the sensitivity is low, in contrast to the HPV DNA testing. Despite the success of these measures, new biomarkers are still considered to aim increasing sensitivity and specificity of screening and diagnosis. Significant alterations in microRNA (miRNA) expression have been detected in several cancers with variable consistency. To investigate the stratification role of miRNAs between normal epithelium and cervical intraepithelial neoplasia (CIN2–3), we screened the expression of 667 miRNAs to identify significant markers (*n* = 10), out of them 9 miRNAs were applied in the study (miR-20b, −24, −26a, −29b, −99a, −100, −147, −212, −515-3p) along with RNU48 and U6 as the references. To benchmark the miRNAs, 22 paired (tumor-free and tumor tissue pairs) laser microdissection-obtained cervical formalin fixed, paraffin embedded tissue samples were assayed. The expression of miR-20b was 2.4 times higher in CIN2–3 samples as compared to normal tissues (*p* < 0.0001). In the HPV16-positive subsets of the samples (*n* = 13), miR-20b showed 2.9-times elevation (*p* < 0.001), whereas miR-515 was 1.15-times downregulated (*p* < 0.05) in CIN2–3 as compared to normal tissue. These results suggest the potential value of miR-20b as a statification biomarker in order to differentiate neoplastic and non-tumorous cases.

## Introduction

Cervical cancer is a common malignant tumor worldwide among females regarding both incidence and mortality, ranking fourth after breast, lung, and colorectal cancer with an estimated 570,000 new cases yearly and 311,000 cancer related deaths in 2018 according to GLOBOCAN sources [1]. There is, however, great variability across the geographic regions of the world, with higher numbers occurring in several developing countries, where cervical cancer ranks second for both incidence and mortality [1]. The effect of population-based screening programs using the Papanicolaou („Pap”) test for cervical cancer resulted a significant decrease both in incidence and mortality [1–3]. Introduction of world-wide accepted guidelines for new evaluation methods, including the one formulated at the Bethesda Conference in 2001 [4], led to further decline in cervical cancer incidence. Discovery of the etiological role of the human papilloma virus (HPV) in cervical carcinogenesis [5] and the introduction of population-based vaccination programs were further steps towards the prevention of cervical cancer [6]. It became clear, however, that while being highly specific, the sensitivity of cytology is relatively low regarding the detection of high-grade squamous intraepithelial lesions (HSIL) or cervical intraepithelial neoplasia (CIN) [7]. Recently, more sensitive though less specific HPV DNA molecular detection tests have been introduced into the screening system effectively reducing cervical cancer incidence better than cytology screening alone. The demonstration of high-risk HPV (hrHPV) infection however does not prove whether the infection is transient or of the transforming type. [6, 8–10]

Several new biomarkers have been applied for detection of transformed cells aiming to increase the sensitivity and/or specificity of the screening tests and/or in combination with cytology and/or with HPV DNA testing [8, 11]. More recently, several groups have demonstrated aberrant expression of microRNAs (miRNAs) in premalignant and malignant cervical lesions, suggesting that this might be used as a more specific biomarker [3, 12–19]. The majority of these works analyzed cervical cancer and/or premalignant lesions, with no surrounding tumor-free tissues coming from the same patient. For this reason, we performed a study comparing the miRNA expressions of HSIL/CIN2–3 lesions and histologically non-altered surrounding cervical epithelium in pairs deriving from the same patients.

## Materials and Methods

### Patients and Specimens

In total, 10 paired (normal/diseased) formalin fixed paraffin embedded (FFPE) histological samples with CIN1, 2, 3 and in situ carcinoma (CIS) were applied for miRNA screening and further 44 FFPE tissues (22 pairs of CIN2–3 and surrounding normal tissue) were chosen for validation of the results. The samples were selected from the archives of the 2nd Department of Pathology of Semmelweis University. The study was performed with the permission of the National Ethical Committee (V-R-021/04346–4/2013), according to the principles of the Declaration of Helsinki and with written consent from the patients. The samples derived form patients between 22 and 45 years of age at the time of obtaining tissue material with an average of 32.9 years (Table 1). Laser captured microdissection was used for selection and isolation of morphologically altered, dysplastic and non-tumorous foci performed by experienced pathologists. The 4–5 μm-thick FFPE sections were cut on FrameSlides 1.4 μm Pet Membrane (MicroDissect GmbH, Herborn, Germany). Leica AS LMD (Meyer Instruments, Houston, Texas, USA) instrument was used for microdissection of the areas of HSIL and non-altered epithelium, which were collected in BRAND^®^PCR-tubes (0,5 ml, BrandTech^®^ Scientific, Merck KGaA, Darmstadt, Germany). A total of 10–12 microdissected sections were collected from the selected areas for the subsequent RNA isolation.

**Table 1:** List of patients

No	Age	HPV type
1	31	16^+^
2	30	16^+^
3	38	Negative
4	25	16+
5	44	16+
6	32	Negative
7	34	33^+^
8	27	16^+^
9	30	Negative
10	32	58^+^
11	23	16^+^
12	25	16^+^; 31^+^; 33^+^
13	35	18+
14	28	16^+^, 66^+^, 45^+^, 59^+^
15	35	16^+^
16	34	Negative
17	43	16^+^,18^+^
18	36	16^+^
19	43	Negative
20	22	16^+^
21	35	LR^+^ and 56^+^
22	42	16^+^
*Average: 32.9*		

*HR: high risk HPV types; LR: low risk HPV types*

### HPV Genotyping Test

The Human papilloma virus (HPV) viral DNA detection was perfomed by CONFIDENCE HPV™ test (GenoID, Budapest, Hungary), which detects HPV16, HPV18 separately, and the other high-risk HPV-types (31, 33, 35, 39, 45, 51, 52, 56, 58, 59, 66 and 68) in group using multiplex real-time polymerase chain reaction technology [20].

### RNA Isolation

Total RNA including the miRNA fraction was extracted from the removed tissue areas by RecoverAll Total Nucleic Acid Isolation Kit (Ambion brand by Life Technologies, Carlsbad, California, USA) according to the manufacturer’s instructions. The purified RNA was kept until use at −80 °C after the determination of the RNA concentration by a NanoDrop 1000 spectrophotometer (Life Technologies of Thermo Fischer Scientific, Waltham, MA, USA).

### miRNA Screening

A preliminary miRNA expression screening was perfomed in 10 paired (normal/tumor) samples using TaqMan Array (TaqMan Array Human MicroRNA Cards Set v2.0, Panel A and B, Life Technologies, Foster City, CA, USA) according to the manufacturer’s instructions (Table 2). The two-card sets contained 667 specific assays to human miRNAs and four control assays (three endogenous control and one negative control assays). In order to increase sensitivity of the analysis for miRNAs being present in lower concentration, a preamplification step was included using Megaplex PreAmp Primers (Applies Biosystems by Life Technologies Foster City, CA, USA). The miRNA measurements were performed in 384-plate format in a 7900HT Fast Real-Time PCR System (Applies Biosystems by Life Technologies Foster City, CA, USA). Ct values were calculated using the SDS software v.2.1 using automatic baseline settings.

**Table 2:** Kits used for preliminary miRNA expression screening

Name of the kit	Catalogue Number
TaqMan MicroRNA Reverse Transcription Kit	4,366,596
Megaplex RT Primers Human Pool A	4,399,966
Megaplex RT Primers Human Pool B	4,399,968
TaqMan PreAmp Master Mix Kit	4,384,267
Megaplex PreAmp Primers Human Pool A	4,399,233
Megaplex PreAmp Primers Human Pool B	4,399,201
TaqMan 2X Universal PCR Master Mix, No AmpErase UNG (1 × 5 ml)	4,324,018
Human Array A and B	4,400,238

### Determination of the Expression Levels of miRNAs Identified in the Screening

The expression of the selected miRNAs listed in Table 3 was determined using TaqMan MicroRNA Assays (Life Technologies brand of Thermo Fisher Scientific Inc). Reverse transcription (RT) and quantitative polymerase chain reaction (qPCR) were performed according to manufacturer’s instructions. RT reaction was carried out using TaqMan MicroRNA Reverse Transcription Kit containing 10 ng total RNA. The qPCR was performed by applying TaqMan Universal Master Mix II, no UNG and 0.65 μL of the RT product. Owing to low copy numbers of miRNAs initially present in the samples, two miRNAs (miR-147 and miR-515) were preamplified just before qPCR for 12 cycles including the assays for reference as well. The amplification reaction was run in triplicates on a LightCycler 480 Instrument II (Roche Diagnostics, Indianapolis, IN, USA). Relative expression (2^∆Cq^, where ΔCq = Cq_Ref_-Cq_miR_) was calculated applying the average of RNU48 and U6 as the most stable reference determined by the NormFinder application [21].

**Table 3:** The list of selected miRNAs

Assay ID	miRNA
001014	hsa-miR-20b
000402	hsa-miR-24
000405	hsa-miR-26a
000413	hsa-miR-29b
000435	hsa-miR-99a
000437	hsa-miR-100
000469	hsa-miR-147
000515	hsa-miR-212
002369	hsa-miR-515-3p
For reference:	
001006	RNU48
001973	U6

### Statistical Analysis

The differences between normal and HSIL/CIN2–3 lesions were analyzed by means of non-parametric Wilcoxon Matched Pairs Test using STATISTICA software, version 12 (StatSoft Inc., Tulsa, OK, USA). A *p* value of 0.05 was set as the threshold for statistical significance.

## Results

To investigate whether miRNAs could predict the difference between normal and HSIL/CIN2–3 tissue samples, we analyzed the expression of 667 miRNAs in a preliminary set of samples (*n* = 10). The paired healthy and diseased samples were evaluated according to the average down-regulation / up-regulation and their respective standard deviation and miRNAs with significant values (compared to the zero change) were selected for the study. It is noteworthy that there were significant average fold changes in the data sets. A total of 52 dysplasia upregulated and 27 dowregulated miRNAs with more than 3-fold changes were detected, but the majority of these changes was also highly variable, which renders them statistically insignificant. The final selection of the miRNAs was based on significantly different expression in diseased as compared to healthy tissue pairs (miR-20b, −24, −26a, −100) and additional miRNAs were selected based on a literature review (miR-29b, −99a, −147, −212, −515-3p).

**Fig. 1:**
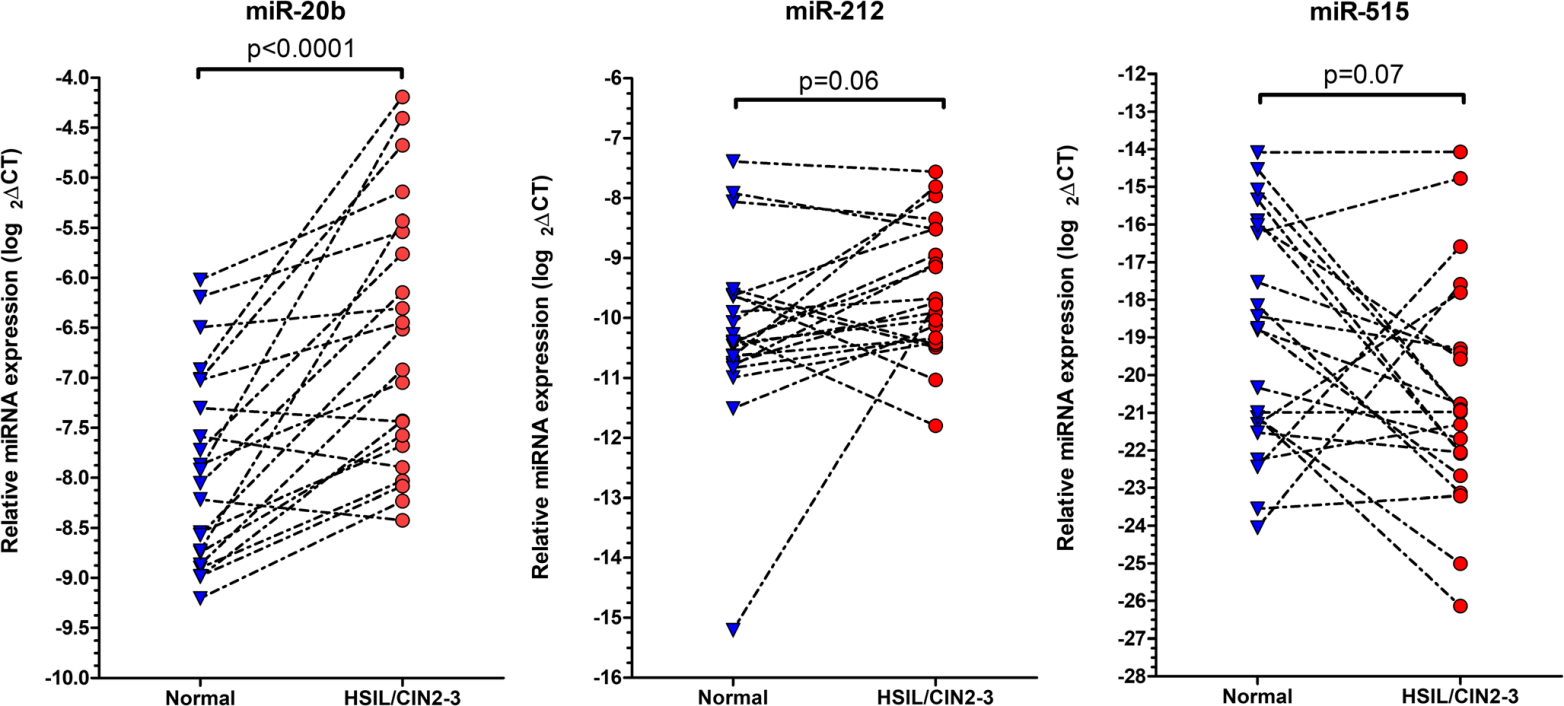
Relative miRNA expression in HSIL/CIN2–3 and corresponding normal cervical samples. Black interrupted lines represent the pairs of samples and indicate the direction of alteration of miRNA expression in dysplastic samples as compared to normal tissue

In order to assess the expression of these selected 9 miRNAs in HSIL/CIN2–3 and surrounding morphologically non-altered (“normal”) epithelium, further 22 paired FFPE cervical samples were used from the patient cohort. Statistical analysis of the miRNA expressions in the sample pairs (normal contra HSIL) showed a 2.4 fold overexpression of miR-20b in HSIL/CIN2–3 samples compared to normal tissues with high statistical significance (*p* < 0.0001) (Fig. 1). Additionally, miR-212 was also elevated with 1.6 fold increase (*p* < 0.06) and miR-515 showed 4 fold downregulation (*p* < 0.07) in dysplasia tissue compared to normal; however, these differencies were not statistically significant (Table 4). The other miRNAs analyzed did not show any difference between dysplasia and normal areas.

**Table 4:** miRNAs showing statistical differences between HSIL/CIN2–3 and corresponding normal tissue

miRNA	p value
In the 22 pairs	
miR-20b	**<0.0001**
miR-212	0.06
miR-515	0.07
In HPV16+ samples	
miR-20b	**<0.001**
miR-515	**<0.05**

**Fig. 2:**
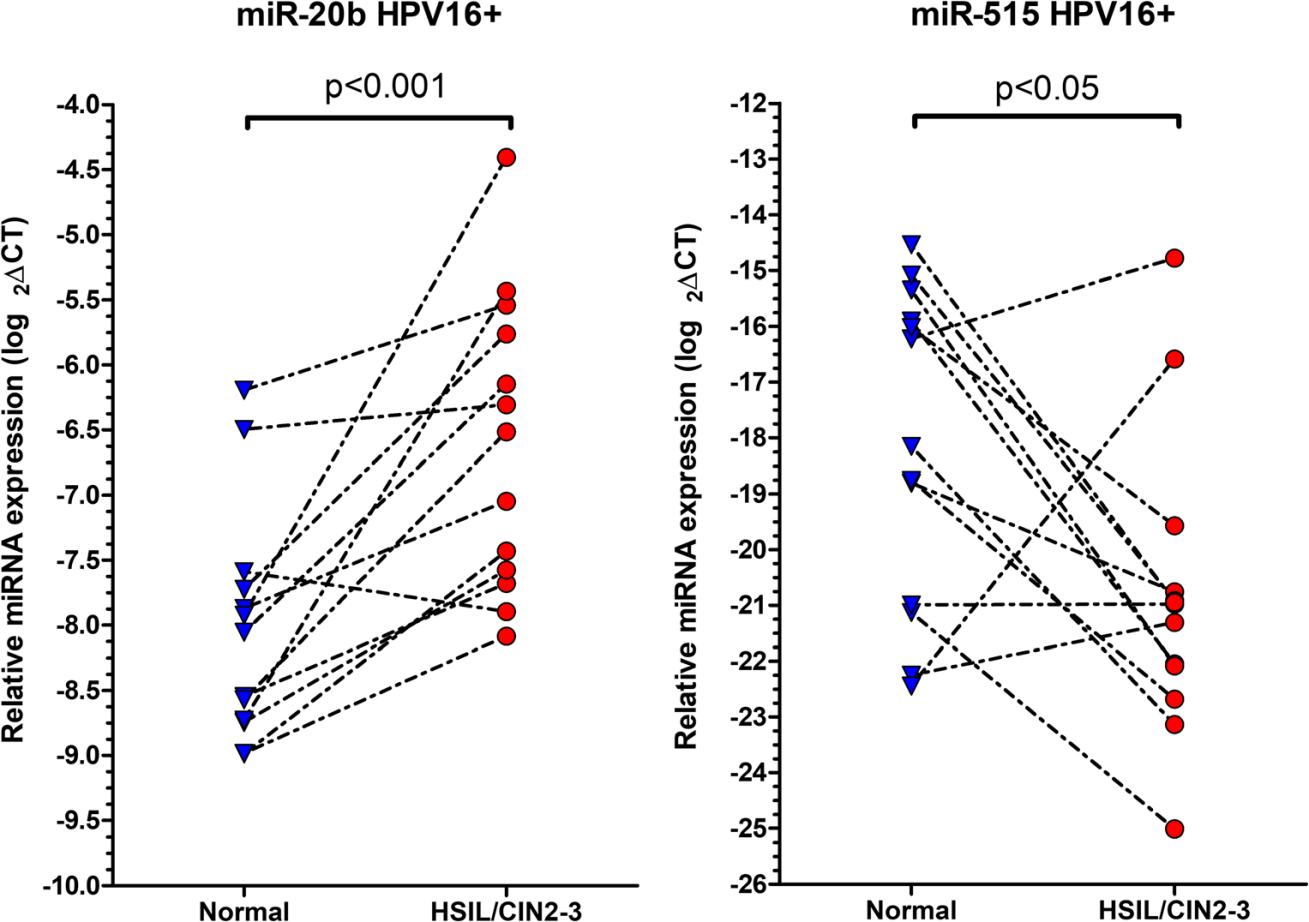
Relative miRNA expression in HSIL/CIN2–3 samples known to be HPV16 positive and in corresponding normal cervical samples (*n* = 13). Black interrupted lines represent the pairs of samples and indicate the direction of alteration of miRNA expression in dysplastic samples as compared to normal tissue

When investigating miRNA expression in the samples tested to be HPV16-positive (*n* = 13), miR-20b showed 2.9 fold upregulation (*p* < 0.001) and miR-515 was 1.15 fold downregulated (*p* < 0.05) in HSIL/CIN2–3 compared to normal tissue, which were statistically significant (Fig. 2).

## Discussion

The novelty of our study is the pairs of HSIL/CIN2–3 and morphologically non-altered tissues, derived from the same patients as the source of miRNAs, precisely removed by laser microdissection, which has been rarely done in previous reports [22]. Our results demonstrated significantly increased expression of miR-20b in HSIL/CIN2–3 lesions in comparison to the surrounding morphologically non-altered cervical epithelium; while increased miR-212 and decreased miR-515 expression were also detected, however, these expressional differences were not statistically significant regarding the whole study population.

miRNAs are a family of small (18–25 nucleotides in length) single-stranded non-coding RNA molecules, which regulate gene expression at post-transcriptional level. miRNAs affect messenger RNAs (mRNAs) by degradation or by repression of translation via binding to the 3′ untranslated region of the targeted mRNAs [11, 17, 23]. Until now, approximately 2500 human miRNAs have been described in the miRBase database (www.mirbase.org), consisting of tissue-, organ-, cell- and „differentiation”- specific miRNAs. It has been shown that dysregulated miRNA-expression is involved in various biological processes including carcinogenesis. miRNAs may act as „oncomirs” functioning as either oncogenes or tumor suppressors depending on the type of tissue and stage of tumor development. miRNAs may be up- or downregulated in cancer tissue and thus these molecules have been suggested to be potential novel tumor markers [3, 13, 14, 17, 19, 24–28].

Accordig to Pardini et al., 24 studies targeting miRNA expression in cervical cancer show „great variability”. The variability, however, can be attributed to the differing numbers of samples (and consequently the varied statistical power) and the studied different histological backgrounds (premalignant or malignant lesions). Nevertheless, the analyzed samples (liquid, solid, FFPE or frozen) and the RNA isolation methods are also varied. Despite the mixed backgrounds, miR-29a and miR-21 were the most often found down- and upregulated miRNAs in invasive cervical cancer, while the altered expression of other miRNAs, especially miR-20b, was found to be associated with a range of patological lesions including from premalignant to malignant stages [17]. Cheung et al., by comparing normal epithelium, CIN and carcinoma samples, found 10 upregulated and 2 downregulated miRNAs in CIN lesions. The upregulation of miR-20b was found to be of the highest fold changes (2.89) in CIN2–3 lesions compared to normal tissue. As miR-20b was shown to be upregulated from the stage of premalignancies to invasive cancer, our results confirm the earlier findings regarding HSIL/CIN2–3 samples [29].

miR20b is encoded by the miR-106a-363 cluster located on human chromosome X and an autoregulatory feedback loop exists between E2F1 and miR-20b-5p, which was shown to be involved in proliferation and differentiation [30]. In a breast cancer study, miR-20b was shown to act as a tumor suppressor by inhibiting the migration and invasion of breast cancer cells [30] . Others demonstrated that HPV E6-regulates miR-20b and promotes invasion in cervical cancer by targeting the tissue inhibitor metalloproteinase 2 [31].

Regarding other dysregulated miRNAs in cervical cancer development, Pardini et al. and He et al. reviewed the available studies, with listing miR-26a, −29a, −99a, −100, and − 212 reported to be downregulated in CIN2–3 and/or cervical cancer in comparison to normal cervical epithelium [17, 32]. In addition, aberrant expression of miR-24 and -29b has been observed in cervical cancer [33–35] and downregulated miR-147 and upregulated miR-515-3p have been found in several solid cancers [36, 37]. However, we did not find statistically significant differences in the expression of these miRNAs when all of the 22 paires of HSIL/CIN2–3 samples were analyzed.

When examinig the miRNA expression in the 10 pairs of preliminary samples, we observed highly variable fold changes with relative large standard deviation, suggesting that the samples were rather heterogenous. This means that samples of different molecular background might have been put into the same group of HSIL/CIN2–3, which signifies the finding of a more efficient way for the stratification of samples with the aim to reduce the heterogeneity between the samples even more. For this reason, we chose a subgroup of samples tested positive for HVP16 and found a more upregulated miR-20b in HPV16-positive HSIL/CIN2–3 samples as compared to normal cervical epithelium. Association between miRNA expression and hrHPV infection has been reported [16, 32, 38]. For example, Wang et al. (2014) detected 13 up- and downregulated miRNAs, which were associated with hrHPV 16 and 18 [39].

In association with CIN2–3 and cervical carcinoma, several further miRNAs showing dysregulated expression have been reported, such as miR-218, 29a, −101, −140-5p [40–44]. In the study of Gocze et al. (2015), progressively increased expression of miR-27a was demonstrated in CIN2–3 as compared to CIN1 and in squamous cell carcinoma (SCC) as compared to CIN2–3, while the levels of miR-34a were found to be lower in CIN2–3 when compared to CIN1 and in SCC in comparison to CIN2–3. These data were found to be correlated with HPV16 positivity [14]. Differences were also detected in miRNA expression between SCC and adenocarcinoma [13]. Overexpression of several miRNAs was shown in SCC (miR-21, −27a, −34a, −155, −196, −203, −221) regardless of HPV status, however miR-21, −27a, −34a, −196a, and − 221 characterized HPV positive SCC, in contrast to adenocarcinoma having the same HPV status [13].

More recently, altered miRNA expression has been reported in non-solid tissues, such as biofluids (blood, vaginal mucus, saliva, urine etc.) [18, 45]. Four miRNAs were upregulated in CIN2–3, SCC and adenocarcinoma, showing a correlaion with hrHPV. The data were confirmed in frozen tissues coming from the same patients and miR-20b was among the upregulated miRNAs, as found in our HSIL/CIN2–3 cases. miRNAs may be associated with „Argonaute (AGO) proteins” or located in extracellular vesicles implying that miRNAs are „diversely distributed” across tissues and extracellular fluids, meaning that miRNAs are present intracellularly and extracellularly as well [45].

To summarize, our study revealed overexpression of miR-20b in HSIL/CIN2–3 tissue lesions as compared to the morphologically non-dysplastic adjacent epithelium. This might suggest the use of miR-20b as a potential biomarker in order to differentiate neoplastic from normal tissues. Further studies may examine the use of miR-20b as a marker in vaginal fluid/mucous and/or in liquid based cytology samples.
